# Separation of gold and other rare materials from an ensemble of heterogeneous particles using a NdFeB magnetic circuit

**DOI:** 10.1038/s41598-019-40618-2

**Published:** 2019-03-08

**Authors:** Chiaki Uyeda, Keiji Hisayoshi, Kentaro Terada

**Affiliations:** Institute of Earth and Space Science, Graduate School of Science, Osaka University, Toyonaka, Osaka, Japan

## Abstract

Most solid particles, composed of diamagnetic or weak paramagnetic materials, cannot be extracted by a conventional magnetic separator. Here we report that an ensemble of heterogeneous particles, composed of bismuth, gold, graphite and rock forming minerals are separated into fractions of different materials by small NdFeB magnetic plates. It is based on a recent finding that acceleration of a translating particle, induced by magnetic volume force in an area of field gradient, is uniquely determined by intrinsic susceptibility of material; the acceleration is independent to particle mass. The setup will serve as an effective technique of pre-treatment in analysing mixture of heterogeneous particles; such a technique is desired in various research fields of science, and the magnetic separation may play a role of a “chromatography technique” conventionally used in the analysis of organic molecules. The portable and low-cost system could provide a breakthrough for on-site research in industrial and medical fields as well as in resource explorations in nature. Extraction of rare materials such as gold or platinum becomes possible in a hazardless manner.

## Introduction

In a conventional magnetic separator, the translation of a particle is induced by an attractive magnetic force caused by a magnetic field gradient; here, the force is generally believed to act only on particles that bear a strong positive magnetization. The induction of a strong magnetic force was realized in a diamagnetic particle by binding a small magnetic bead to the particle; the technique was extended to develop *in vivo* drug delivery systems^1^. A separation using- a field gradient force was realized as well in magnetized DNA samples^2^. Levitation was realized on a human fingertip using a field gradient produced by a small NdFeB magnetic block^3^. It is seen from these attempts that the demand for inducing effective magnetic forces on weak magnetic (i.e. diamagnetic and paramagnetic) particles is potentially large, because the technique can dynamically translate “magnetically inert” particles by a relatively simple machine. Accordingly, various attempts were made to dynamically control weak magnetic materials by introducing strong field generators^4–7^.

In a recent study, a mixture of particles composed of various weak magnetic materials was magnetically separated into groups of different materials with no magnetic attachments on the particles^8^; here, the separation was caused by a field gradient produced by a NdFeB magnetic circuit, and the particles were able to translate through a diffused area by applying a microgravity (*μg*) condition. The mechanism of the above separation was expected based on an energy conservation rule recently proposed in a particle that translated by a magnetic volume force^9,10^. It was experimentally confirmed that acceleration of the particle induced by volume force in an area of field gradient is determined by intrinsic magnetic susceptibility of material, and was independent to its mass. The results were in contrast to the common convention that weak magnetic particles are magnetically inert, especially in the low field intensity produced by a permanent magnet. However, the efficiency of the material separation of the reported system was not high enough for practical use because the quantity of sample that could be separated in a single turn of a *μg* experiment was less than 0.001 g, and it took more than 15 minutes to complete a single *μg* experiment^8^. It was also necessary to introduce a drop-shaft system (50 × 50 × 180 cm) to apply the *μg* condition^8–10^, which was not useful for practical applications.

In the present report, the separation of weak magnetic particles was performed for the first time in terrestrial gravity conditions using a facile NdFeB circuit; the system is simple and inexpensive compared to the previous setup using the *μg* condition^8^. The possibility of improving the resolution of the separation using the present apparatus is discussed. The final goal of the improvement is to separate and identify most materials that appear in investigating mixtures of solid particles, which is often necessary in a pre-treatment process of a refined material analysis.

## Methods

**Figure 1:**
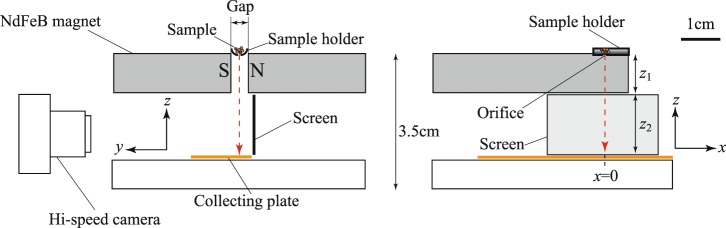
Sectional view of an apparatus used to conduct the magnetic separation of various diamagnetic and paramagnetic particles by a pair of FeNdB permanent magnets. The field intensity monotonically decreases in the *x*-direction, and the maximum field at the centre is 9.6 kG, which was measured by a gaussmeter. The locus observed in the translating particles recorded by the hi-vision camera is shown in Fig. 2 and the particles recovered on the collecting plate are shown in Fig. 3. The magnetic susceptibility of the particles ranged between −50 × 10^−7^ to +340 × 10^−7^ emu/g (see Table 1), which means that the apparatus is capable of separating most existing materials when the separation resolution is improved.

As shown in Fig. 1, the size of the apparatus developed to separate the weak magnetic particles was less than 10 cm in length, and its weight was below 1000 g. Two NdFeB plates (4 × 4 × 1 cm) were used to compose a magnetic circuit that produced a monotonically decreasing field along the x-axis located in a gap (0.4 cm in width) between the two plates. The mixture of the particles was maintained in a sample holder with a half-piped scape (0.4 cm in diameter and 0.2 cm in depth). Before the experiment, the orifice (*ϕ* 0.07 cm) at the bottom of the sample holder was set just below the top level of the gap. A collecting plate was set parallel to the *xy*-plane at the bottom of the translating area. A section paper was attached to the plate to measure the horizontal separation, *x*_T_, of the individual particles with respect to the position of the orifice; the positions were measured after they were collected on the plate.

The numerical data for the five materials observed in the present study are listed in Table 1. The paramagnetic olivine sample was a product from San Carlos, New Mexico^8–10^, while the pyroxene sample was a product of Tanzania. The paramagnetic susceptibility, *χ*_PARA_, of the above samples was measured by the vibrating sample magnetometer (VSM), and the results are listed in Table 1. The values were consistent with their Fe concentrations determined by a chemical analysis. In most of the materials that exist in nature, the concentration of magnetic ions is below the level of San Carlos olivine. The three diamagnetic samples, namely, bismuth, graphite and gold, were cut from synthetic blocks with high purities (>99.99%) by using a titanium knife to avoid contaminations of small ferromagnetic particles. Among the popular solid materials, graphite is known to have the largest |*χ*_DIA_| value^11^; accordingly, most of the *χ* values reported for the existing weak magnetic materials in nature overlap with the *χ* values of the five materials. The diamagnetic susceptibilities of the three materials measured by the VSM method were consistent with the published values. The particles were manually pushed towards the orifice position one by one using a thin copper wire and were dropped from the orifice with small initial velocity; it took approximately 20 seconds to complete the translation of the particles set in the sample-holder (~30 particles). The time-dependent photographs of the particle motions were recorded by a high-speed camera^8–10^ from the *y*-axis direction.

**Table 1:** Numerical data of the sample particles observed in the present study.

Materials	published^11^ *χ*	horizontal separation *x*_T_	*δx*_T_	*v*_T_	*χ* obtained from *v*_T_
	(×10^−7^ emu/g)	(cm)	(cm)	(cm/s)	(×10^−7^ emu/g)
Graphite	−52	1.00	0.1	10.5 ± 0.6	36 ± 13
Bismuth	−13	0.20	0.17	5.5 ± 0.7	14 ± 5.0
Gold	−1.42	−0.02	0.04	—	—
Pyroxene	105	−0.37	0.18	—	—
Olivine	340	−1.06	0.16	—	—

## Results

**Figure 2:**
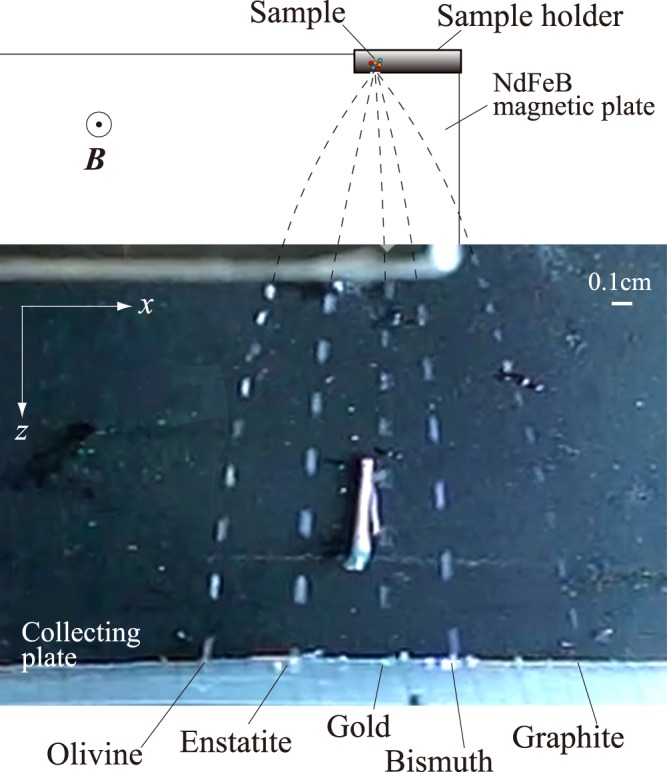
A serial photograph of the translating particles taken by a high-speed camera from the direction of the *y*-axis in Fig. 1. The interval of the image is 0.0033 s.

Figure 2 shows the locus of five particles observed by the high-speed camera during their translation. The particles are composed of five different materials that have different *χ* values, as shown in Table 1. It can be seen from the locus that the material separation proceeds due to the variance in the horizontal velocity induced in the individual materials. As mentioned before, the range of *χ* values of the five materials overlaps with the range of all the *χ* values reported for the existing materials, and it may be concluded that the compact NdFeB circuit used in the present study has the potential to separate an ensemble of heterogeneous particles existing in nature. The published *χ* values of some popular materials^11^ are listed in Table 2 for comparison; the variances of these values are derived from the variances in the electron distributions that exist between the materials^8^, indicating that an intrinsic *χ*_DIA_ value is assigned to a solid material.

**Table 2:** Diamagnetic susceptibility, *χ*_DIA_, of solid materials^11^.

Materials	*χ*_DIA_ (×10^−7^ emu/g)
Anthracene	−8.19
Bismuth	−9.8
Calcite	−3.6
Cellulose	−4.2
Corundum	−3.63
Diamond	−5.8
Forsterite	−3.3
Gold	−1.42
Graphite	−52
Indium	−1.12
MgO	−2.56
Naphtalene	−7.08
Platinum	+9.8
Pyroxene	−4.0
Quartz	−3.7
Salt	−5.2
SiC	−4.27
Silver	−1.92
Tin	−2.2

**Figure 3:**
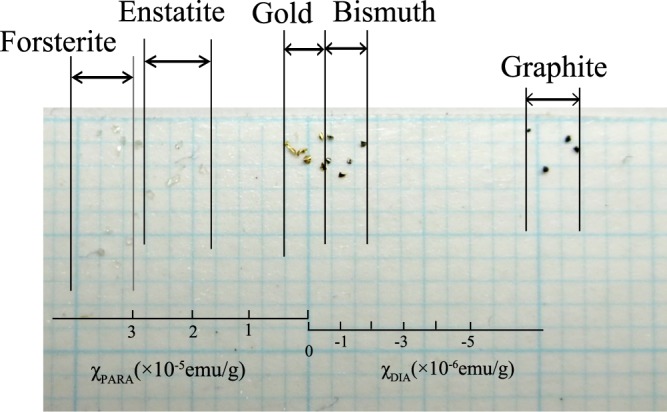
Photograph of the collecting plate after the translation definitely showing the material separation of all the particles. The scale in the lower portion show the approximate *χ* values that are expected in the particles collected at individual positions. As described in the text, horizontal acceleration of a particle is induced by a field-gradient force during its translation though the gap of magnetic circuit, which is followed by another translation between the gap and the plate. The approximate *x*_T_ value can be calculated as a sum of the horizontal translations in the above two areas. Numerical relationship between *χ* and *x*_T_ were calculated to obtain the scale; detail of the calculation is described in Supplementary Note B.

As seen in Fig. 3, the particles released from the orifice are generally preserved on the collecting plate as different groups of materials which were separated during the translation. The length of the horizontal translation *x*_T_ of each material with respect to the position of the orifice is shown in Table 1; here, the values show the average of the separations observed for several particles of a single material. The sequence of the *x*_T_ value is consistent with that of the published *χ* values listed in Table 1, confirming the efficiency of the separating system.

In previous studies on field-induced translation observed in weak magnetic particles^8–10^, the *χ*_DIA_ value per unit mass of the particle was obtained from their translating velocity, and it was proposed that the material of the particle could be identified by comparing the observed value with the list of published *χ*_DIA_ data^11^. Here, the value was obtained from an energy conservation rule assuming that the magnetic potential of the particle at the initial position [½*mχ*_DIA_*B*_0_^2^] was completely converted to kinetic energy [½*mv*_T_^2^] in the area of *B*~0. Here, *B*_0_ describes the field intensity at the initial position, whereas *v*_T_ is the terminal velocity of the particle in the area of *B*~0; *m* denotes the mass of the particle. Accordingly, *χ*_DIA_ should satisfy the relation,

1 $${\chi }_{{\rm{DIA}}}={v}_{{\rm{T}}}^{2}{B}_{0}^{-2}.$$

In the abovementioned conservation rule, the velocity of the particle at the initial position, denoted *v*_0_, is assumed to be negligibly small. By inserting in Eq.(1) the values of *B*_0_ and *v*_T_ observed in the present experiment, the *χ*_DIA_ values of graphite and bismuth are calculated as (39 ± 10) × 10^−7^ emu/g and (11 ± 5.0) × 10^−7^ emu/g, respectively. Here, the field intensity at the orifice was observed as 5.3 ± 1 kG and used as the *B*_0_ value in the above calculation; here temperature *T* was 298 K. The time-dependent velocities of the graphite and bismuth particles in the *x*- and *z*-directions were obtained from the locus data as shown in Fig. 2, and the numerical values in the *x*-direction were used to obtain the *v*_T_ values (Supplementary Note A). The *χ*_DIA_ values obtained in the above manner agreed fairly well with the published values listed in Table 1, and it may be concluded that the observed translations followed the energy conservation rule (Supplementary Note B). The above consistency of *χ*_DIA_ values show that level of ferromagnetic contamination of the sample particles that may happen during the experimental process is negligible. In the case of paramagnetic materials, the particles translated towards the field-centre area by an attractive field gradient force (see Figs 2 and 3), and it was difficult to quantitatively examine the efficiency of the energy conservation rule, because the profile of the field distribution inside the small area of the circuit gap was difficult to obtain by a standard gaussmeter (Supplementary Note C).

Approximate *χ* values that are expected in the particles collected at individual positions on the collecting plate are shown by a linier scale in Fig. 3. The values are estimated from a field-gradient force, *mχB*(d*B*/d*x*), that is applied on the particle as it translates through the gap of the circuit; detail of calculation is described in Fig. 3 (Supplementary Note B). It is seen that the ranges of particle position observed in individual materials are consistent with the positions expected from the *χ* values assigned to the material in a semi-quantitative manner. The deviations between the expected and actual positions derive from the rough assumption of field distribution inside the gap that was made in the calculation; as mentioned before, it was difficult to obtain a precise field distribution inside the narrow gap. By minimizing the above deviations (Supplementary Note C), material of an unidentified particle can be estimated by comparing the *χ* value on the scale with a list of published values (i.e. Table 2); the estimation is easily performed without consuming sample.

## Discussion

The separating rate (i.e. sample weight per second) is considerably improved in the present apparatus compared to the experiment recently operated in *μg* conditions^8^, because it can continuously separate the particles in open air under normal gravity conditions. The portable size of the setup is useful in various on-site activities, such as geological, biological or industrial field research. Furthermore, the machine is applicable in performing the above activities at the surfaces of various solid bodies in the solar system, because the compact size and rigid structure of the setup is suitable for a remote sensing mission^8^. The results described in Figs 2 and 3 provide a firm proof that the simple principle of separation deduced from eq.(1) is effective for existing materials. In the case of separating micron-size particles, the dimensions of the machine can be decreased even more because the separation is realized in a field area with a reduced size. It is noted that temperature may vary from about 200 K to 380 K when the system is operated in various field activities on the earth; this variance of temperature would considerably alter the field intensity produced by a neodymium magnet to change the *B*_0_ value of the experiment. Therefore it is necessary to calibrate the *B*_0_ value according to the experimental temperature in the individual experiments to obtain the precise *χ* values assigned to the individual particle.

It is seen in Table 1 and in Fig. 3 that the standard deviation of the measured *x*_T_ values for the same material, denoted *δx*_T_, is considerably large, which directly disturbs the efficiency in separating two materials with different *χ*_DIA_ values. In the actual experiments, the *v*_0_ value of the particle at the time of release was not negligible because collisions between the particles may occur as they pass through the orifice. The Coulomb forces caused between the static electricity on the particles surface (as well as those between the particles and the sample stage) could also induce a finite amount of *v*_0_; note that *δx*_T_ was reduced to a certain level in the present study by discharging the particles and the sample stage using an ionizer, however *δx*_T_ was not reduced to a negligible level. The above two factors directly induce *δx*_T_, because *δx*_T_ correlates with *v*_0_ following a relationship *δx*_T_~*v*_0_*T*_f_; here *T*_f_ denote the duration of free fall.

As shown in Table 2, an intrinsic *χ*_DIA_ value is assigned to a solid material, and the numerical values indicate that the materials of two different particles are generally distinguished if the variance of their measured *χ*_DIA_ values (denoted *δχ*) is above the level of ~10^−8^ emu/g^11^. In contrast, the dispersions observed in Fig. 3 for bismuth graphite and gold particles indicate that the separation of the two materials by their positions is difficult when *δχ* is at the level of 10^−7^ emu/g. To improve the separation efficiency, the above-mentioned disturbances caused by particle collision and/or Coulomb forces should be diminished to minimize *v*_0_ of individual particles. Specifically, *δx*_T_ values of two materials in Fig. 3 should be reduced to a level that is smaller than the variance of *x*_T_ between the two materials, defined hereafter as *Δx*_T._

It is noted that the efficiency of the separation (& material identification) is also improved by increasing the variance of horizontal velocity, *Δv*_T_, between the two materials, which directly increases the *Δx*_T_ between the materials. A direct way to increase *Δv*_T_ is to enhance the magnetic force produced in the magnetic circuit. Such circuits with enhanced field gradients were previously introduced to detect the *χ*_DIA_ values of small particles from their field-induced translations^9^, and the field gradient produced by the circuit was as large as 6250 G/cm. The above experiment was conducted in a *μg* facility that required a limited pay load size, and the circuit with an edge length of ~10 cm (weight: ~1.5 × 10^3^ g) suited this condition; it is noted that this circuit is suitable for on-site transportation in a material separation project in normal gravity condition (Supplementary Note D).

The extraction & identification of new solid phases from a heterogeneous particle ensemble may lead to important discoveries in various researches based on material analysis. Although it is possible to perform refined survey on a mixture of the heterogeneous particle using various microprobe devices, it is difficult to conclude by these devices whether or not the minor particles included in the sample are thoroughly identified without omission, and new categories of minor material phases may remain undiscovered in the mixture^8^. In such cases, it is desirable to separate the particles into groups of different materials before performing the refined analyses. In this sense, the proposed method can be used as a new type of “chromatography” technique^12^ specialized for particle mixture samples.

Resource exploration of rare metallic materials such as alluvial gold is a possible eligible application using this NdFeB magnet separation. So far, mercury amalgamation has been used for gold/silver extraction from parent rocks, and pure gold/silver is collected by evaporating the mercury in chemical plants, causing various hazards (e.g. health hazards, pollution in the atmosphere, and mercurial toxic waste). As seen in Table 2, the *χ*-value of Au is −1.42 × 10^−7^ emu/g, which is distinct from that of silver (−1.9 × 10^−7^ emu/g), paramagnetic pyrite and other major minerals in the parent rocks that possess paramagnetic susceptibility. Hence, gold particles can be collected after a “physical” crashing without any chemical treatment that may cause the various hazard problems, as mentioned above. Moreover, the extraction of gold particles from urban mining resources is also applicable because the |*χ*| values of the resin^11^ materials used as the electronic circuit boards are much larger (>3 × 10^−7^ emu/g) than that of gold. Extraction is also possible for other rare materials such as indium (*x* = −1.1 × 10^−7^ emu/g), platinum (*χ* = + 9.8 × 10^−7^ emu/g) or niobium bearing particles in a hazardless manner from both natural and urban resources.

Attempts have been recently made to decontaminate the soils polluted with radioactivity using the strong field-gradient force produced by a superconducting magnet after the Tohoku earthquake^13^. Here the magnetic force that acts on the soil particles is considerably enhanced by coating paramagnetic ions on the particle surface. Previous studies were efficient, but a facile magnetic-separation system has been desirable for on-site examination and decontamination over a wide area. Hence, the proposed method is also expected to promote the on-site separation of polluted soil dusts.

Another advantage of the proposed separation system is that the sample particles are preserved in the course of separation & identification. The present study proved that this facile NdFeB magnet circuit is applicable for precious samples, such as the asteroid regoliths collected by the Hayabusa2^14,15^ and OSIRIS-Rex sample return mission^16,17^, which are mixtures of various minerals and organics. By improving the efficiency of the proposed method, even micron-order grains can be thoroughly separated by the proposed principle, and their material can be identified without sample loss. Using a conventional analyser, such as a mass spectrometer, it is difficult to precisely identify the material of a solid particle without consuming sample.

In conclusion, we have established a new process to separate and/or identify most existing solid materials from *χ* = −52 × 10^−7^ emu/g (diamagnetic graphite) up to + 340 × 10^−7^ emu/g (paramagnetic olivine) by a rigid pocket-size system operated in open air, which is assembled and operated at a low cost (<10^5^ JPY). Further improvements to optimize the parameters of *δx*_T_ and *Δv*_T_, which directly contribute to the resolution of separation and identification, would result in a breakthrough in various research fields of science, both in on-site field activities as well as in pre-treatment processes of refined analyses in laboratory. Finally, the mass independent property observed in a translated particle, which is presently recognized only in gravitational motions, will be easily induced by the cause of magnetic volume forces in outer space; it may be commonly observed in nature as well because both solid particles and magnetic field co-exist in various regions of the galaxy^8^. The mass independent property of field-induced translation has been recently confirmed in particles that bear ferro- (feri-) magnetic moment^18^ and the proposed separation is effective in all categories of magnetic material that exist in outer space.

## Supplementary information

Supplementary Note
