# Supplementary material for: Separation of gold and other rare materials from an ensemble of heterogeneous particles using a NdFeB magnetic circuit
Source: Sci Rep. 2019 Mar 8;9:3971. doi: 10.1038/s41598-019-40618-2 (PMC6408432; doi:10.1038/s41598-019-40618-2)
Supplement: Supplementary file 1 — Supplementary Note [file 41598_2019_40618_MOESM1_ESM.docx]

**Separation of gold and other rare materials from an ensemble of heterogeneous particles using a NdFeB magnetic circuit**

*C. Uyeda, K. Terada, K. Hisayoshi

Institute of Earth and Space Science, Graduate School of Science, Osaka University

(*Correspondence to [uyeda@ess.sci.osaka-u.ac.jp](mailto:uyeda@ess.sci.osaka-u.ac.jp))

**Supplementary information**

Supplementary Note A

The observed velocity along the *z*-axis followed the free-fall relationship, *v*(*t*)_z_ = 979.8 *t*; i.e. the duration of the free fall, 0.072 s**,** was consistent with the length of the free fall that was 2.54 cm. The experimental values of horizontal velocity *v*(*t*)*_x_* of the particles conversed to a constant value in an area of *B*~0.

Supplementary Note B

A horizontal acceleration *a*(*χ*) of the particle during its translation through the circuit gap is described as *a*(*χ*) = *χB*(d*B*/d*x*) [dyn/g]. Based on the field distribution that was measured inside the narrow gap, *B*(d*B*/d*x*) is assumed as 1.16×10^7^ [G^2^/cm] at *x* < 0, and 8.85×10^6^ at *x >* 0 [G^2^/cm]. Numerical value of *x*_T_(*χ*) is calculated as *x*_T_ (*χ*) = *a*(*χ*)(½*t*_1_^2^+ *t*_1_*t*_2_) =(2.52 ± 0.38)×10^4^*χ*[cm] where *t*_1_ denote the duration inside the gap, whereas *t*_2_ is the duration outside the gap; they are calculated as t_1_=0.043 s and *t*_2_=0.029 s according to the vertical heights described in Fig. 1, namely *z*_1_=0.9 cm and *z*_2_ =1.55 cm; the value of t_2_ is consist with the time observed from Fig. 2. The uncertainty of the *x*_T_ (*χ*) values in the above equation is mainly attributed to the low spatial resolution of *B* measurement that was performed inside the narrow gap.

Supplementary Note C

Although the main purpose of this study is to realize separation of existing materials by using the smallest circuit that fulfil the purpose, it is also important to examine the efficiency of the conservation rule on the paramagnetic materials in future studies; this can be done by introducing a circuit with an enhanced gap width. Precise field-distribution inside the gap is required to examine and improve the efficiency of the present system.

Supplementary Note D

The compact magnetic-circuit composed of small NdFeB plates, as described in Fig.1, has been introduced in a number of studies on weak magnetic particles, namely to observe their alignments or translations induced by a static field. In these experiments, it was necessary to set the field generator in a limited area of volume. In the present study, the positions of the small NdFeB plates were optimized through trial & error experiments to realize translations of different particles having a wide range of *χ* values (-50×10^-7^~+340×10^-7^emu/g); accordingly, separation of existing materials using the facile circuit was realized in normal gravity condition.
